# The Induction of a Permissive Environment to Promote T Cell Immune Evasion in Acute Myeloid Leukemia: The Metabolic Perspective

**DOI:** 10.3389/fonc.2019.01166

**Published:** 2019-11-06

**Authors:** Dimitrios Mougiakakos

**Affiliations:** Department of Medicine 5, Hematology and Medical Oncology, Friedrich Alexander University Erlangen-Nuremberg, Erlangen, Germany

**Keywords:** AML—acute myeloid leukemia, immunoescape mechanisms, tumor metabolism, immunotherapy, microenvironment

## Abstract

Acute myeloid leukemia (AML) is the acute leukemia with highest incidence amongst adults. Despite significant improvements in understanding the genomic landscape and the introduction of novel drugs, long-term outcome remains unsatisfactory. Recently, immunotherapeutic approaches have heralded a new era in cancer treatment. The success of allogeneic hematopoietic stem cell transplantation in AML highlights the disease's immunoresponsiveness. Several immunotherapeutic applications are currently under clinical evaluation and include immune checkpoint blockades, T cell-engaging antibodies, and genetically engineered T cells. However, immunoevasive mechanisms employed by AML blasts severely hamper our endeavors. A better understanding of the underlying mechanisms remains a prerequisite for improving treatment efficacy. One of the hallmarks of the cancer cells is metabolic reprogramming, introduced by Otto Warburg's seminal studies during the beginnings of the last century. Nowadays, it is well established that metabolic adaptation is not just an epiphenomenon during oncogenesis but rather a necessity for tumor development and progression. Furthermore, accumulating data suggest an important role of aberrant tumor cell metabolism for immune escape. AML blasts display a number of metabolic alterations that could be linked to immunoregulation, and these include competition over substrates, abundant release of bioactive metabolites, and an overall microenvironmental metabolic re-modeling that favors the induction or survival of immunoregulatory cell subsets such as regulatory T cells. In this review, we outline the immunoevasive character of the AML blasts' bioenergetics, set it into context with oncogenic mutations, and discuss potentially suitable countermeasures and their limitations.

## Introduction

Acute myeloid leukemia (AML) represents the most common form of acute leukemia in adults. Despite advances in AML therapy, treatment outcome remains unsatisfactory. Immunotherapy has heralded a new era in solid and liquid malignancies. Successful usage of allogeneic hematopoietic stem cell transplantation for curing AML suggests its immunoresponsive nature (1). Several immunotherapeutic approaches are currently under clinical investigation, including multispecific T cell-engaging antibodies (2, 3), immune checkpoint blockades, and genetically engineered T cells (4, 5). However, clinical efficacy of immunotherapies is substantially hampered by AML-associated immune escape strategies. Increasing evidence suggests that the cancer cells' hallmark metabolic reprogramming (6) generates a permissive environment. AML blasts display various metabolic alterations, which we will discuss in this review together with their role in relation to immunoevasion and potential counterstrategies.

## The Immunological Side of the Warburg Effect (in AML)

**Figure 1:**
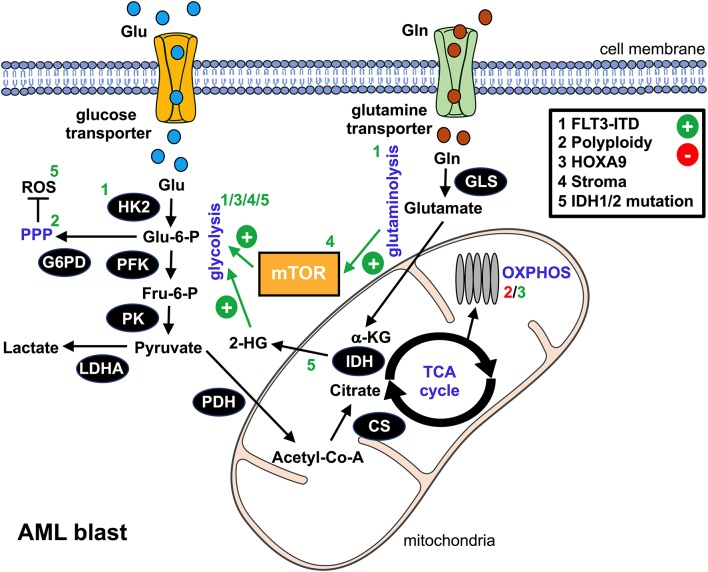
Metabolic alterations in AML blasts. This schematic overview summarizes the bioenergetic rewiring in AML blasts. The black cycles represent key metabolic enzymes. Identified genetic aberrations and/or microenvironmental components that promote (+, green) or suppress (-, red) metabolic pathways in AML blasts are numbered 1–5 and can be found in the upper right box. Affected pathways, metabolic products, or enzymes are labeled with the according number. glu, glucose; gln, glutamine; glu-6-P, glucose-6-phosphate; fru-6-P, fructose-6-phosphate; α-KG, α-ketoglutarate; 2-HG, 2-hydroxyglutarate; ROS, reactive oxygen species; HK2, hexokinase 2; PFK, phosphofructokinase; G6PD, glucose-6-phosphate dehydrogenase; PK, pyruvate kinase; LDHA, lactate dehydrogenase A; PDH, pyruvate dehydrogenase; CS, citrate synthase; IDH, isocitrate dehydrogenase; GLS, glutaminase; mTOR, mammalian target of rapamycin; PPP, pentose phosphate pathway; TCA, tricarboxylic acid cycle; OXPHOS, oxidative phosphorylation.

To date, it is well established that malignant cells consume high levels of glucose that they preferentially ferment to lactic acid even in the presence of oxygen and a fully competent mitochondrial oxidative phosphorylation (OXPHOS). This phenomenon was first described by Otto Warburg in the 1920s (7) and is known as the “Warburg effect.” As of yet, numerous functions of the “Warburg effect” have been proposed (8). The rate of adenosine triphosphate (ATP) production per unit glucose is 18 times lower for aerobic glycolysis as compared to respiration. However, the absolute amount of ATP at any given time point is similar due to the 10–100 times faster kinetics of aerobic glycolysis (9), which might give aberrant cells a selective advantage when competing over limited substrates (e.g., in a hypoxic environment such as the bone marrow) (10). Furthermore, increased glycolytic flux delivers carbon sources for anabolic processes (i.e., the *de novo* synthesis of nucleotides, lipids, and proteins) required to meet the biosynthetic demands of highly proliferative cells such as AML blasts (8). Another suggested role of aerobic glycolysis is to maintain the intracellular redox homeostasis by, amongst other things, allowing the increased biosynthesis of reducing equivalents *via* the pentose phosphate pathway (PPP). AML blasts display elevated levels of reactive oxygen species (ROS) (11) and would largely benefit from enhanced compensatory antioxidative machinery since moderate ROS levels can drive the disease, whereas higher ROS levels can result in cell death (12). In addition, a proportion of glucose is directed into the hexosamine biosynthesis pathway and promotes protein glycosylation, which is involved in maintaining high levels of the anti-apoptotic Mcl-1 (13). Similarly to numerous other malignant entities, aerobic glycolysis is also found increased in AML (as compared to physiological hematopoietic cells) when analyzing primary blasts and AML-derived cell lines or when performing metabolic imaging of the bone marrow niche (14–16) (Figure 1). Moreover, data suggest that glycolytic activity of AML blasts at diagnosis (a panel of six serum metabolites involved in glucose metabolism) and expression levels of key glycolytic molecules such as pyruvate dehydrogenase kinase can be of (negative) prognostic value for AML (14, 15, 17). Differences between the distinct WHO AML subtypes were not observed, suggesting effects independent from the cytogenetic-based risk stratification. Anecdotal reports describe clinically relevant Warburg effect-triggered systemic alterations (i.e., hypoglycemia together with lactic acidosis) in patients with AML (18).

Several oncogenic pathways such as HIF-1α or c-Myc have been linked to the neoplasia's glycolytic switch. In AML, an internal tandem duplication (ITD) within the Fms-like tyrosine kinase (FLT3/ITD) represents an unfavorable genetic aberration. Recent findings suggest that FLT3/ITD promotes aerobic glycolysis through an AKT-mediated upregulation of the glycolytic pacemaker enzyme hexokinase 2 (HK2) (19). Polyploidy, which can be found in most types of cancer, is also linked to enhanced glycolysis in AML, most likely due to an activation of PPP while directly repressing OXPHOS (20). Homeobox (HOX) genes, in particular HOXA9, are overexpressed in a substantial proportion of AML cases. They drive the upregulation of the cells' glycolytic program via demethylases, such as the jumonji C containing H3K9 demethylase (JMJD1C), thus contributing to an aggressive phenotype (21). Several of the aforementioned signaling pathways yield an (over-)stimulation of the mammalian target of rapamycin (mTOR), which acts as a master regulator of cellular bioenergetics and which is consequently active in AML (22). In addition to cell-intrinsic processes, microenvironmental crosstalk such as interaction with mesenchymal stromal cells or stromal cell-derived factor 1 (SDF1) can trigger an mTOR-dependent metabolic rewiring toward aerobic glycolysis (23, 24).

Such AML-mediated glucose deprivation could substantially impact the functional competence of various immune cells that utilize glucose as an energetic substrate, including monocytes, NK cells, dendritic cells, and, in particular, T cells, which display an exhausted and senescent phenotype in AML patients (25).

Naïve T cells meet most of their energetic demands by use of OXPHOS of fatty acids. However, triggering the T cell receptor (TCR) in conjunction with CD28 co-stimulation leads to an mTOR-orchestrated (26) rapid upregulation of aerobic glycolysis that enables clonal expansion and distinct T cell effector functions such as the production of INF-γ (via posttranscriptional regulation) (27, 28). Consequently, culturing T cells under glucose-deprived conditions severely impedes TCR downstream signaling, proliferation, and cytokine production, leading to an exhausted-like state (29, 30). Accordingly, highly glycolytic tumors display an increased resilience toward adoptive T cell therapy approaches while previous blocking of glycolysis enhances the antitumor activity of subsequently transferred tumor-reactive T cells (31). Consequently, bolstering the T cells' glycolytic competence by, for example, overexpressing phosphoenolpyruvate carboxykinase 1 has improved their tumoricidal activities in preclinical models (32).

In addition to depleting glucose, enhanced aerobic glycolysis leads to an abundant production of lactic acid, thereby shifting the overall pH. High levels of lactic acid have an inhibitory effect on human T cells, resulting in reduced cell activation, proliferation, and effector functions (33). This was recently validated in murine tumor models (34). Neutralizing the lactic acid-induced acidosis by bicarbonate application has improved the efficacy of immune checkpoint blockades as well as of adoptively transferred T cells in preclinical settings (35). Taken together, the blunting of T cell responses in animal tumor models has been attributed to both lactic acid accumulation (31) and the metabolic competition of glucose (36). Interestingly, T cell-suppressive regulatory T cells (T_Regs_), which are considered important contributors to tumor-induced immunoevasion (37) and accumulate in the peripheral blood and the bone marrow of AML patients (38), display enhanced resilience toward lactic acid while mainly relying on OXPHOS (and not glycolysis) (39). Collectively, these observations suggest that the milieu generated by aerobic glycolysis performed by malignant cells (and mimicking inflammatory-like conditions) skews the balance between T cell-immunoreactivity and immunotolerance toward the latter one.

In close resemblance to effector T cells, NK cells switch toward aerobic glycolysis upon activation in a mTOR-dependent fashion (40). This metabolic shift represents a prerequisite for NK cells to exert their tumoricidal functions (41). As anticipated, high glycolytic activity in tumor tissues blunts NK-cell responses *via* acidification (42). It remains to be elucidated, however, whether and how glucose depletion might affect NK-cell function *in vivo*.

## Reactive Oxygen Species

As previously exemplified for lactic acid, bioactive metabolites can be of an immunoregulatory impact. One very well-studied phenomenon is oxidative stress. This metabolic condition results from the accumulation of so-called reactive oxygen species (ROS) such as superoxide or hydrogen peroxide. Those short-lived molecules are hyperpermeable and highly reactive. Oxidative stress is typically found in cancer patients (43). High ROS levels negatively impact TCR signaling, T/NK cell activation, and viability (44, 45). Interestingly, T_Regs_ appear more resilient toward ROS-mediated toxicity by, amongst other things, releasing the antioxidant thioredoxin-1 (46, 47). This further corroborates the notion that the tumor microenvironment not only supports the induction of immunoregulatory cell subsets but also endows them with survival advantages over their immunoreactive counterparts. Oxidative stress is present in AML patients and can correlate with the risk for disease relapse (12, 48). Most studies suggest that the constitutive activation of nicotinamide adenine dinucleotide phosphate oxidase-2 (NOX2) (48) is the primary source of AML blast-derived ROS (i.e., superoxide), with mitochondrial ROS-production linked to OXPHOS playing a secondary role (49). Those free radicals inactivate antileukemic T/NK cells by triggering PARP-1-dependent apoptosis, thereby contributing to immunoevasion (50).

Interestingly, histamine dihydrochloride (HDC) can efficiently reduce the NOX2-dependent ROS formation by triggering the histamine type 2 receptor that is expressed on myeloid cells including AML blasts (51). The ability of HDC to shield tumor-reactive lymphocytes represents a vital basis for clinical trials testing the combination of HDC (as an indirect antioxidant) with (T/NK cell-stimulating) low-dose interleukin-2 (IL-2) (52). Leukemia-free survival was found to be improved, leading to the approval of HDC and IL-2 as a maintenance strategy and *post-hoc* analyses revealing that patients with myelomonocytic or monocytic AML might benefit most (50, 53).

## 2-hydroxyglutarate: a Novel Immunoregulatory Onco-Metabolite

Increased D-2-hydroxyglutatarate (2-HG) serum levels were recently identified as a novel negative prognostic marker for AML (54). 2-HG abundance has been mainly attributed to somatic heterozygous mutations in genes encoding for isocitrate dehydrogenase 1 (IDH1) and its mitochondrial homolog, IDH2 (Figure 1). These mutations initially identified in gliomas are found in up to 20% of all newly diagnosed AMLs, especially in cases with normal cytogenetics, as well as in premalignant proliferative diseases such as myelodysplastic syndrome (55).

IDH enzymes convert isocitrate into α-ketoglutarate (α-KG). Beyond its role as an intermediate of the Krebs cycle, α-KG represents a co-substrate for a number of metabolic partners, including >60 mammalian dioxygenases and demethylases. Mutations occur at critical arginine residues of the enzymes' active site (R132 in IDH1 and R140/172 in IDH2). The amino acid substitution prevents its normal catalytic function (“loss-of-function”) and at the same time confers a neomorphic enzymatic activity that facilitates reduction of α-KG to 2-HG (“gain-of-function”). The rate of 2-HG production far exceeds the rate of homeostatic clearance, leading to pathological 2-HG accumulations.

Increasing evidence suggests that 2-HG acts as an “onco-metabolite,” driving proliferation and differentiation arrest. Notably, 2-HG and α-KG are structurally similar, except that the oxygen atom linked to C2 in α-KG is replaced by a hydroxyl group in 2-HG. This structural similarity suggests that 2-HG might exert its oncogenic effects through the competitive inhibition of α-KG-dependent enzymes (56). Exposure to high levels of 2-HG inhibits histone demethylase JMJD1C, thereby altering the cells' epigenetic profiles and resulting in hypermethylation, which represents a hallmark of myeloid malignancies and premalignant disorders. Furthermore, 2-HG leads to an allosteric inhibition of prolyl hydroxylases, which normally downregulate hypoxia-inducible factor 1α (HIF-1α). 2-HG-mediated HIF-1α stabilization could thereby contribute to the malignant cell's “pseudohypoxic” response (”Warburg” effect), as recently observed in tumors carrying mutated IDH1 (leading to glucose depletion together with lactic acid accumulation) (57). An additional metabolic alteration linked to 2-HG overproduction is oxidative stress, which can exert AML blast-promoting effects (58) and at the same time hamper immunosurveillance. Here, redox homeostasis is disrupted by an increased consumption of NAPDH during 2-HG synthesis, which, amongst other functions, acts as an indirect antioxidant (59).

In addition to promoting a pro-glycolytic and ROS-enriched environment, recent studies indicate that 2-HG might directly impact T cell responses. It has been shown that T cells are capable of efficiently taking up 2-HG, further validated by 2-HG-enriched T cells being exclusively found in samples from patients with IDH-mutated AML (60). In the context of gliomas, it was further shown that IDH mutations and high 2-HG levels lead to a reduced T cell activation, proliferation, and migration, consequently resulting in lower T cell infiltration at the tumor site (61). Inhibitory effects of 2-HG were mediated by interference with ATP-dependent TCR signaling and the calcium-dependent transcriptional activity of nuclear factor of activated T cells (NFAT) downstream of the TCR. Blocking IDH activity has improved the efficacy of peptide vaccination approaches in preclinical glioma models (62). Overall, reported data on immune-related effects of 2-HG in AML remain limited, and now that pharmacological IDH inhibitors have been introduced in AML treatment, it will be of great interest to evaluate their impact on the patients' immune function.

## Tackling Amino Acids

**Figure 2:**
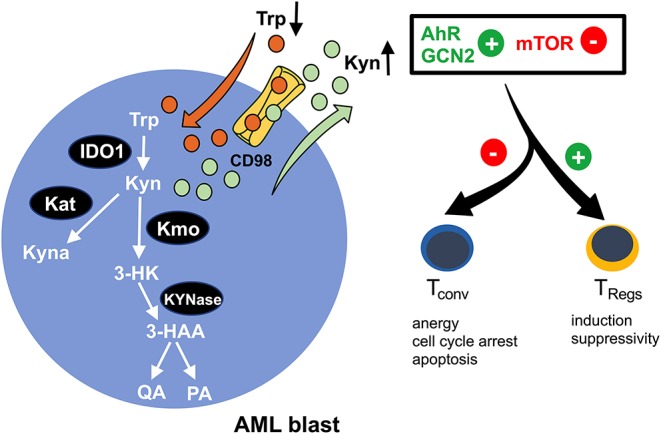
Enhanced tryptophan-turnover in AML blasts. Increased expression of indoleamine-2,3 dioxygenase 1 (IDO1) in AML blasts leads to tryptophan (Trp) depletion. It is catabolized to kynurenine (Kyn), resulting in extracellular kyn accumulation. A proportion of kyn is intracellularly converted to kynurenic acid (Kyna) by kyn aminotransferase (Kat) or to 3-hydroxy kyn (3-HK) by kyn 3-monooxygenase (Kmo). Subsequently, 3-HK is further processed into 3-hydroxanthranilic acid (3-HAA) by KYNase, which is further converted into quinolinic acid (QA) and picolinic acid (PA). The microenvironmental shortage of Trp and simultaneous abundance of Kyn promote activation of the aryl-hydrocarbon receptor (AhR) and the non-derepressing 2 protein kinase (GCN2) and suppress the mammalian target of the rapamycin (mTOR) pathway, thus skewing immune responses away from immunoreactivity (by impeding conventional T cells) toward immunotolerance (by reinforcing regulatory T cells/T_Regs_).

Indoleamine-2,3-dioxygenase (IDO) is a *bona fide* representative of metabolic enzymes that exerts dual effects (in terms of immunological impact) by simultaneously depleting essential substrates and producing bioactive metabolites (Figure 2). It catalyzes oxidation of tryptophan (trp) into kynurenine (kyn) and can be found to be expressed in immune cells such as macrophages and in a variety of malignant tissues, including ovarian cancer, melanoma, or head and neck cancer (63). Tryptophan degradation can be assessed *ex vivo* by measuring tryptophan and kynurenine levels. In fact, a highly increased kyn/trp ratio in AML patient sera indicates an enhanced IDO-activity while negatively correlating with overall survival (64, 65). Furthermore, it has been shown that in >50% of the cases tested at diagnosis, AML blasts constitutively express IDO as potentially being (co-) responsible for the observed systemic (aforementioned metabolic) effects (66). In addition to this, AML blasts are capable of inducing IDO^+^ bystander cells, such as myeloid derived suppressor cells (MDSCs) (2). IDO-mediated shortage of trp and the accumulation of kyn lead to T cell anergy, proliferation arrest in the G_1_ cell cycle phase, and apoptosis (67). The underlying mechanism lies in the activation of the non-derepressing 2 protein kinase (GCN2) (67) and suppression of mTOR signaling (68), which is triggered by tryptophan depletion and further enhanced by kyn binding to the aryl-hydrocarbon receptor (AhR) (69). Furthermore, it has been reported that AML blasts promote formation of T cell-suppressive T_Regs_ (from conventional T cells) in an IDO-mediated fashion, further potentiating a tolerogenic environment (70). Again, GCN2 and AhR activation have both been implicated in driving the induction of T_Regs_ and their immunosuppressive capacity (67, 69) (Figure 2). A phase 1b/2a trial (ClinicalTrials.gov identifier: NCT02835729) evaluating the IDO inhibitor indoximod as part of the maintenance regimen after standard induction and consolidation chemotherapy is currently ongoing.

In addition to trp, arginine (arg), a non-essential amino acid, plays an important role in regulating immune responses (71). T cells respond toward arg deprivation with autophagy, CD3ζ chain downregulation, and apoptosis. AML blasts express and secrete arginine-catabolizing arginase II, whereas arginase I is only detected at low levels (72). The arginase II in the patients' plasma is significantly higher as compared to healthy donor-derived samples. Consequently, arg serum concentrations are lower in AML patients (73). Culturing T cells in the presence of AML patients' plasma reduced their proliferative response toward activating stimuli, which could be restored by arg repletion (72). Furthermore, AML blasts promote an arginase-mediated repolarization of macrophages toward an immunosuppressive type 2 phenotype. Inhibition of arginase activity leads to an enhanced *in vitro* cytotoxicity of antigen-specific and chimeric antigen receptor (CAR) T cells against AML blasts (73).

Glutamine (gln) represents a key carbon source fueling OXPHOS in AML blasts, thus supporting their rapid expansion (Figure 1). In fact, intracellular gln levels positively regulate mTOR activity. Inhibition of gln uptake by the SLC1A5 transporter or of its conversion to glutamate by glutaminase both cause proliferation arrest and apoptosis of AML blasts without affecting conventional CD34^+^ hematopoietic progenitors (74, 75). In fact, the glutaminase inhibitor CB-839 synergizes with Bcl2 inhibitors (75). Moreover, targeting the FLT3 kinase inhibits glycolysis (as mentioned previously) while rendering AML cells dependent on gln (76). At the same time, a number of studies suggest an important role of gln for proper T cell function, including proliferation and cytokine production (77). However, the exact impact of an *in vivo* competition over gln on AML-directed T cell responses needs to be further elucidated based on recent data, which shows that transient gln restriction might even favor the formation of cytotoxic T cells together with antitumor immunity (78, 79).

## Immunometabolic Countermeasures

Based on the well-established interconnection between tumor metabolism and its impact on the immunometabolic fitness of T cells, several metabolic pathways are already being assessed in clinical and preclinical studies. Targeting mTOR as a metabolic master regulator could represent an obvious choice in AML (22, 23). However, the role of mTOR for T cell metabolism should be kept in mind and might explain mixed results in terms of promoting (80) vs. inhibiting (81) T cell functions in different cancer models. Inhibitors of drivers of oncogenic signaling that also control metabolic features such as mutated FLT3 or IDH are currently under therapeutic exploitation and it will be of great interest to study their impact on intrinsic (anti-AML) immunity. In fact, treating AML patients with the FLT3 inhibitor midostaurin has led to a T_Reg_ reduction (82).

Interfering with immunological checkpoints could also represent a strategy for restoring metabolic T cell competence. Constitutive and inducible programmed death ligand 1 (PD-L1) is found on AML blasts (83) while patient-derived T cells display an increased expression of its cognate receptor programmed cell death protein 1 (PD-1) (84). Interfering with the PD-L1/PD-1 crosstalk enhanced anti-AML immunosurveillance in murine models and boosted the *in vitro* efficacy of CD33/CD3 bispecific antibodies (3, 85). Furthermore, reports suggest that signaling via PD-1 impedes glycolysis in T cells and myeloid cells, contributing to functional deficits (86). Immune checkpoint blockades could reinvigorate T cell metabolism, but the issue of substrate deprivation (of e.g., glucose, trp, and arg) would still remain, substantially affecting efficacy. At this point, combining immune checkpoint inhibitors (or other immunotherapeutics) with a direct metabolic interference could be a promising approach. IDO and arginase inhibitors are, in fact, currently under clinical evaluation for AML. In terms of blocking the Warburg effect, compounds such as 2-deoxy-D-glucose display antileukemic activity (13), but their off-target impact on T cell metabolism (and consequently their effector functions) needs to be taken into consideration since similar anti-glycolytic approaches have, for example, been successfully tested in T cell-driven autoimmune disease models; these show an amelioration of symptoms partially due to inhibition of T cell metabolism (87).

The introduction of adoptive cell transfer concepts in AML, including TCR-gene transduced (5) and chimeric antigen receptor (CAR) (4) T cells, allows us an *ex vivo* T cell re-modeling with the aim of achieving superior resilience toward detrimental microenvironmental cues (e.g., oxidative stress) and of enhancing metabolic fitness. Cytokines decisively regulate T cell metabolism; culturing T cells in the presence of IL-15 has shown (in contrast to IL-2) to drive mitochondrial biogenesis, skewing bioenergetic dependency away from aerobic glycolysis and toward fatty acid oxidation (FAO). I has also shown to endow them with an increased antioxidant capacity (88), which translated in better *in vitro* and *in vivo* antitumor activity (89). In addition, compounds such as the mitochondrial fusion promoter Mdivi are currently tested in preclinical models (90), convincingly showing that direct metabolic reprogramming holds the potential to improve adoptive cell therapies.

Going one step further, genetic engineering could be utilized for metabolically bolstering T cells before being adoptively transferred. Proof of concept studies have been carried out with TCR-transduced and CAR T cells that overexpress the key antioxidant catalase (91, 92). In addition, CAR construct design has been shown recently to determine the T cells' metabolic profiles. CAR T cells carrying a CD28 signaling domain preferentially perform aerobic glycolysis, while 4-1BBζ CAR T cells meet their energetic demands *via* FAO (93). These 4-1BB-triggered metabolic adaptations are paralleled by enhanced mitochondrial biogenesis, spared respiratory capacity (and thereby better metabolic adaptability), and memory cell formation, which is in line with recent reports suggesting that 4-1BB promotes *in vivo* CAR T cell longevity (94).

## Conclusion

**Figure 3:**
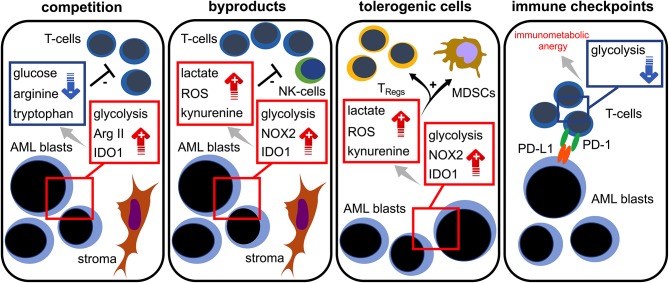
Immunometabolic interplay in AML. Increased glycolytic activity, expression of arginase II (Arg II) and indoleamine-2,3-dioxygenase 1 (IDO1) in AML blasts lead to glucose, tryptophan, and arginine depletion; these are required for proper T cell functionality (= competition). Stromal cells are capable of further triggering those metabolic pathways. Aerobic glycolysis, NADPH-oxidase 2 (NOX2) activity, and IDO1 in AML blasts abundantly produce bioactive metabolites (= waste products) such as lactate, reactive oxygen species (ROS), and kynurenine that hamper T cell responses. Increased levels of lactate, ROS, and kynurenine lead to a preferential survival and/or induction of regulatory T cells (T_Regs_) and the induction of myeloid derived suppressor cells (MDSCs) (= tolerogenic cells). The PD-L1 expression of AML blasts (= immune checkpoint) could cause a state of immunometabolic anergy in T cells by binding its cognate receptor PD-1.

Taken together, increasing evidence suggest an intimate link between the AML blasts' bioenergetics and T cell immunity. Taking into consideration the current emergence of immune-based therapeutic approaches (in AML), which include immune checkpoint blockade, T cell-engaging multispecific antibodies, and genetically modified T cells, it is essential to mechanistically understand the immunometabolic crosstalk for developing the means to improve T cell function. As of today, several mechanisms promoting immunometabolic escape have been described for AML: competition (with immune cells) over critical nutrients such as glucose or amino acids, increased metabolic byproducts such as ROS that negatively impact immune function, microenvironmental metabolic remodeling that endows immunoregulatory subsets (such as T_Regs_) with survival advantages, and expression of checkpoint ligands that impair the immune cells' metabolic competence, such as the ability to utilize certain nutrients (Figure 3). For the future it will be important to shape the AML milieu into one that is more favorable for T cells and to combine immunotherapies with metabolic interventions. Importantly, similarities between AML and T cell metabolism should be kept in mind in order to prevent potential counterproductive off-target effects. Using such combinations in a well-thought-out manner may enable the improvement of modern AML therapy.

## Author Contributions

The author confirms being the sole contributor of this work and has approved it for publication.

### Conflict of Interest

The author declares that the research was conducted in the absence of any commercial or financial relationships that could be construed as a potential conflict of interest.
